# Supplementary material for: What does it take to learn the rules of RNA base pairing? A lot less than you may think
Source: bioRxiv. 2025 Aug 2:2025.07.31.668042. Preprint. [Version 1] doi: 10.1101/2025.07.31.668042 (PMC12324431; doi:10.1101/2025.07.31.668042)

Generic legend

base pair annotations
- covarying mutations
- compatible mutations
- no mutations observed

nucleotide present
97% 75%
90% 50%

nucleotide identity
N 97%
N 90%
N 75%

—?— possible stem

connector (zero length)
variable-length region
variable-length loop
variable-length stem
variable-length stem-loop
modular sub-structure
modular hairpin

R = A or G. Y = C or U. "nt" = nucleotides. "P1" = pairing element 1. "SD" = Shine-Dalgarno (predicted ribosome-binding site). "start" = start codon.

Example with pseudoknot and modular structure

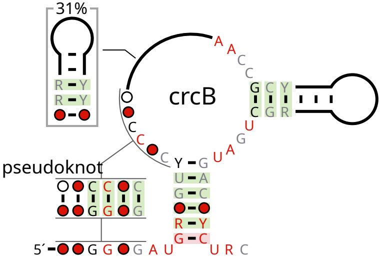

Putting "pseudoknot" over the connector

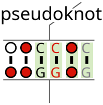

Example with labels for stem, SD & start

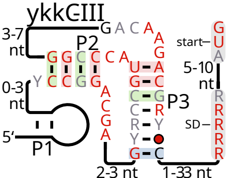

Supplement: Supplement 1 [file media-1.gz › supplemental_material/src/rscape_v2.6.0.tar.gz!/rscape_v2.6.0/lib/R2R/R2R-current/build-package/R2R-1.0.6.cand7-1/generic-annotations/generic-annotations.pdf]
